# An index for measuring functional extension and evenness in trait space

**DOI:** 10.1002/ece3.7577

**Published:** 2021-05-06

**Authors:** Tao Zhang, Grant M. Domke, Matthew B. Russell, Jeremy W. Lichstein

**Affiliations:** ^1^ Department of Forest Resources University of Minnesota St. Paul MN USA; ^2^ Northern Research Station USDA Forest Service St. Paul MN USA; ^3^ Department of Biology University of Florida Gainesville FL USA

**Keywords:** community assembly processes, cumulative distribution function, functional diversity index, minimum spanning tree, null model, species pool, species richness, trait space

## Abstract

Most existing functional diversity indices focus on a single facet of functional diversity. Although these indices are useful for quantifying specific aspects of functional diversity, they often present some conceptual or practical limitations in estimating functional diversity. Here, we present a new functional extension and evenness (FEE) index that encompasses two important aspects of functional diversity. This new index is based on the straightforward notion that a community has high diversity when its species are distant from each other in trait space. The index quantifies functional diversity by evaluating the overall extension of species traits and the interspecific differences of a species assemblage in trait space. The concept of minimum spanning tree (MST) of points was adopted to obtain the essential distribution properties for a species assembly in trait space. We combined the total length of MST branches (extension) and the variation of branch lengths (evenness) into a raw FEE_0_ metric and then translated FEE_0_ to a species richness‐independent FEE index using a null model approach. We assessed the properties of FEE and used multiple approaches to evaluate its performance. The results show that the FEE index performs well in quantifying functional diversity and presents the following desired properties: (a) It allows a fair comparison of functional diversity across different species richness levels; (b) it preserves the essence of single‐facet indices while overcoming some of their limitations; (c) it standardizes comparisons among communities by taking into consideration the trait space of the shared species pool; and (d) it has the potential to distinguish among different community assembly processes. With these attributes, we suggest that the FEE index is a promising metric to inform biodiversity conservation policy and management, especially in applications at large spatial and/or temporal scales.

## Graphical abstract

We presented a new functional diversity index based on the straightforward notion that a community has high diversity when its species are distant from each other in trait space. This index presents four desired properties: allowing a fair comparison of functional diversity across different species richness levels; preserving the essence of single‐facet indices while overcoming some of their limitations; standardizing comparisons among communities by taking into consideration the trait space of the shared species pool; and having the potential to distinguish among different community assembly processes.

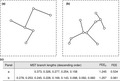

## 1 INTRODUCTION

One of the major tasks in ecology is to advance our understanding about the impact of community structure and diversity on ecosystem functioning (Schneider et al., 2017). Classical taxonomic biodiversity indices focus on species richness and abundance (or evenness) (Pavoine & Bonsall, 2011; Schleuter et al., 2010) and usually ignore the difference among individuals and/or species in their effects on ecosystem functioning (Magurran, 2004; Mouchet et al., 2010). In the past few decades, studies on biodiversity have shown a trend toward incorporating the concept of functional diversity with the realization that ecosystem function and vulnerability are dependent not simply on the number of species, but also on the diversity of functional traits (Díaz & Cabido 2001; Cadotte et al., 2011; Gagic et al., 2015). Various approaches to measuring functional diversity have been developed or proposed in the literature (reviewed in Gagic et al., 2015; Schleuter et al., 2010; Schmera et al., 2017).

Functional diversity indices are typically based on one or more traits of the species in a community, and thus, functional diversity is also sometimes referred to as “trait diversity” (e.g., Fontana et al., 2016; Pavoine & Bonsall, 2011). A species assemblage can be represented by a cloud of points in a multidimensional trait space, where each dimension represents one of the functional traits of interest. Functional diversity indices can be thought of as metrics that quantify different aspects of the distribution of these points in trait space. Most existing indices focus on a single facet of this distribution. In particular, many recent developments and applications of functional diversity indices focus on one of three aspects of the species distribution in trait space: functional richness, evenness, or divergence (Mason et al., 2005; Villéger et al., 2008; Fontana et al., 2016; Carmona et al., 2016; Schneider et al., 2017; Ebeling et al., 2018). Functional richness measures how much trait space is occupied; functional evenness describes the regularity of the abundances of different trait values in the occupied trait space; and functional divergence estimates the degree to which the abundance distribution differs from a centralized pattern (Mason et al., 2005).

Although many indices have been proposed to quantify these facets of functional diversity (Schleuter et al., 2010), these indices often have conceptual and/or practical limitations (Legras et al., 2019; Pardo et al., 2017; Pavoine & Bonsall, 2011; Podani, 2009). For example, many functional diversity indices are intrinsically correlated with taxonomic diversity (e.g., species richness) and are not independent of each other (Cadotte et al., 2011; Schleuter et al., 2010); many indices focus only on the community of interest without reference to a species pool or regional context (Pavoine & Bonsall, 2011); some indices do not perform well in detecting underlying community assembly processes (Botta‐Dukát & Czúcz, 2016; Podani, 2009); some functional richness indices do not take abundance into account and/or are hard to standardize (Laliberté et al., 2014); thus they might be too sensitive to rare species and/or difficult to compare across communities; and some popular indices provide counterintuitive inferences in some cases (Figure S1). Furthermore, some indices can only be calculated for communities with certain characteristics. For example, indices that are based on the community's minimum convex hull in trait space can only be calculated if species richness is greater than the dimensionality of the trait space (Villéger et al., 2008); some functional evenness indices can only be applied to communities containing three or more species (Laliberté et al., 2014); and some functional divergence indices can only be applied to trait spaces of two or more dimensions (Laliberté et al., 2014 ).

The above limitations highlight the need to explore new functional diversity indices with more flexible and desirable properties. Functional diversity is multifaceted, and it may not be possible to design a single index that provides a complete description of functional diversity (Pavoine et al., 2013). Nevertheless, combining more than one facet into a single index, while also addressing some of the conceptual and technical limitations listed above, could simplify and improve some analyses. Two facets that may be usefully combined are the overall trait space that a community occupies (extension) and the variation of interspecific distances in trait space (evenness). For example, two communities with the same extension (e.g., the same range in a one‐dimensional trait space) but different evenness clearly differ in their overall coverage of the trait space, as do two communities with the same evenness but different extension. While it may be desirable to analyze these facets of diversity separately in some applications (Mason et al., 2005; Pavoine & Bonsall, 2011), a combined index may offer advantages in others (Mouchet et al., 2010).

In this paper, we introduce and evaluate a new combined functional extension and evenness (FEE) index. The index is based on a simple notion that a functionally diverse community is composed of species, which are distant from each other in trait space. We show that FEE performs well relative to widely used single‐facet indices in providing intuitive and logically consistent results for previously published test scenarios and in identifying community assembly processes. It also overcomes limitations of some existing indices. For example, FEE has no restrictions regarding the number of species or traits, and it can be applied to abundance or presence–absence data. In addition, FEE is intrinsically independent of species richness because it uses a null model approach to remove the effect of species richness (Mason et al., 2013; Pavoine et al., 2013; Schleuter et al., 2010). Finally, by quantifying functional diversity of communities relative to a common regional species pool, FEE allows for fair comparisons of functional diversity across communities with different species richness levels. We first present the conceptual and technical description of the FEE index, and then, we present multiple tests to explore its properties, including (a) relationships between FEE, species richness, and widely used functional diversity indices; (b) FEE's performance with respect to criteria previously suggested for functional diversity indices (Mason et al., 2003; Ricotta, 2005); and (c) using simulations to evaluate FEE's capacity to diagnose different community assembly processes (neutral, environmental niche filtering, and limiting similarity).

## 2 MATERIALS AND METHODS

### 2.1 Overview

We conceptualize a species assemblage as a point cloud in a multidimensional trait space, where each dimension corresponds to a trait of interest. Our framework can be applied at any scale, and we use the term “community” for convenience to refer to a local community, landscape, region, etc., depending on the relevant scale of a given analysis. The aim of our proposed functional extension and evenness (FEE) index is to quantify the overall spread of species in trait space (extension) and the variation of interspecific distances in trait space (evenness). Our approach is based on three key notions: (a) the unit hypercube representing the trait space of the entire species pool; (b) quantifying trait extension and evenness of the species assemblage using a combined metric of functional diversity; and (c) comparing the observed functional diversity metric to that of random species assemblages of the same species richness. We now describe these three notions in turn and then introduce how we evaluated the FEE index.

### 2.2 The unit‐hypercube trait space of the species pool

We consider the species pool to include all species that are relevant for a given analysis. The species pool is important in our framework because its trait distribution determines how the trait space axes are scaled for all communities in a given analysis (see below), and because it provides the sampling distribution for quantifying the cumulative distribution function of our index (see Equation 2 below and associated text). Depending on the goals of a given analysis, the species pool could be defined as the combined list of species occurring in all studied communities, or the species pool could include not only the species in the studied communities, but also any additional species known to occur in the region of interest. If there are *N* species in the pool, *M* traits are measured for each species, and each trait is normalized to the 0–1 range, then the species can be represented as *N* points in an *M*‐dimensional unit‐hypercube (the trait space). A community of species richness *n* can be represented in this hypercube by a subset of the *N* points (if *n* < *N*) or by all *N* points (if *n* = *N*). The unit‐hypercube trait space collapses to a unit segment in one‐trait cases (*M* = 1) and a unit square in two‐trait cases (*M* = 2). We do not assume any particular normalization method to convert traits to the 0–1 scale. A simple approach is to transform a raw trait value *x* to a normalized value *x*′ according to *x*′ = (*x* − min)/(max − min), where “min” and “max” are, respectively, the minimum and maximum values of a given trait in the species pool. Our framework could accommodate alternative transformations, as long as each community is located within the unit hypercube.

### 2.3 Measuring extension and evenness

**FIGURE 1:**
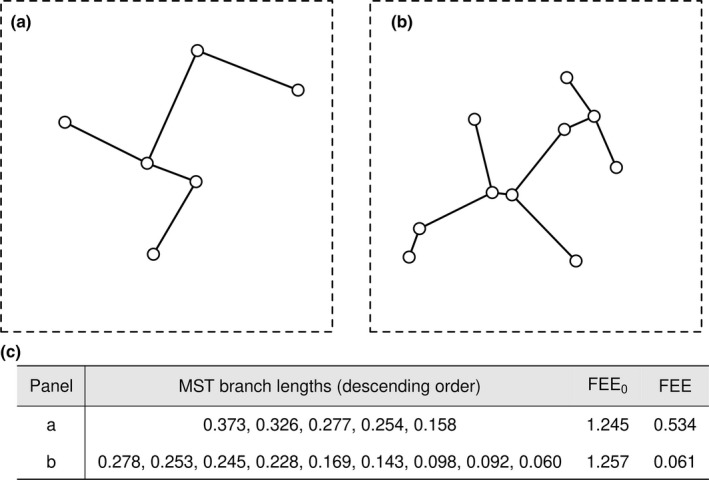
Examples of minimum spanning tree (MST) in a two‐dimensional trait space. A 6‐species assemblage (a) and a 10‐species assemblage (b). The dashed box in each panel indicates the two‐dimensional trait space (unit square). The solid segments are MST branches. Panel c shows branch lengths (i.e., ***l*** in Equation 1), FEE_0_ (Equation 1), and FEE (Equation 2) for the examples in panels a and b

As explained above, a community with *n* species is equivalent to *n* points in trait space. Accordingly, different functional diversity indices can be viewed as quantifying different aspects of the distribution of *n* points in trait space. We characterize this point distribution based on its minimum spanning tree (MST), defined as the tree that connects all points in a multidimensional space while minimizing the sum of branch lengths (as illustrated in Figure 1 here and figure 1c in Villéger et al., 2008). Because of its sensitivity to the spatial distribution patterns of points, MST has been widely used in different scientific fields varying from physics and cosmology to neuroscience and urban science (Naidoo, 2019; Smit et al., 2016; Wu et al., 2018), as well as in evaluating functional diversity (e.g., Schneider et al., 2017; Villéger et al., 2008). We used the “mst” function in the “ape” R package (Paradis et al., 2020) to calculate the MST of a species assemblage in trait space.

To measure the overall trait variation in a community, we consider two aspects of the MST: the total MST length (which represents the overall trait extension in a community) and the proportion of the total MST length comprised by each branch (i.e., evenness: how the total amount of trait difference in a community is partitioned into interspecific trait differences). Our raw metric of functional diversity for a community of species richness *n* is:

(1) $${\text{FEE}}_{0}=\text{sum}\left(l\right)\cdot \sum_{i=1}^{n‐1}\text{min}\left(\frac{l_{i}}{\text{sum}\left(l\right)},\frac{1}{n‐1}\right)=\sum_{i=1}^{n‐1}\text{min}\left(l_{i},\bar{l}\right)$$

where l is the vector of MST branch lengths for the community (l has n‐1 elements); $l_{i}$ is the *i*th branch element in l; and $\bar{l}$ is the mean length of the branches. As the simpler form of Equation (1) (far right) shows, FEE_0_ is the sum of smaller lengths between the raw MST branches and their mean. Two properties of FEE_0_ are that it cannot exceed the total length of MST branches; and FEE_0_ reaches a maximum for a given total branch length when the MST is comprised of equal‐length branches. In addition, FEE_0_ is equivalent to the product of two components, as seen in the more complex form of Equation (1). The $\text{sum}\left(l\right)$ component reflects the overall extension level of the MST in trait space. Similar to the notion of FEve (Villéger et al., 2008), the $\sum_{i=1}^{n‐1}\text{min}\left(\frac{l_{i}}{\text{sum}\left(l\right)},\frac{1}{n‐1}\right)$ component measures the evenness of MST branches by comparing them against the most even case where all branches are equal in length (this component attains a maximum of one when the MST itself is comprised of equal‐length branches).

### 2.4 Calculating the Functional Extension and Evenness (FEE) index

**FIGURE 2:**
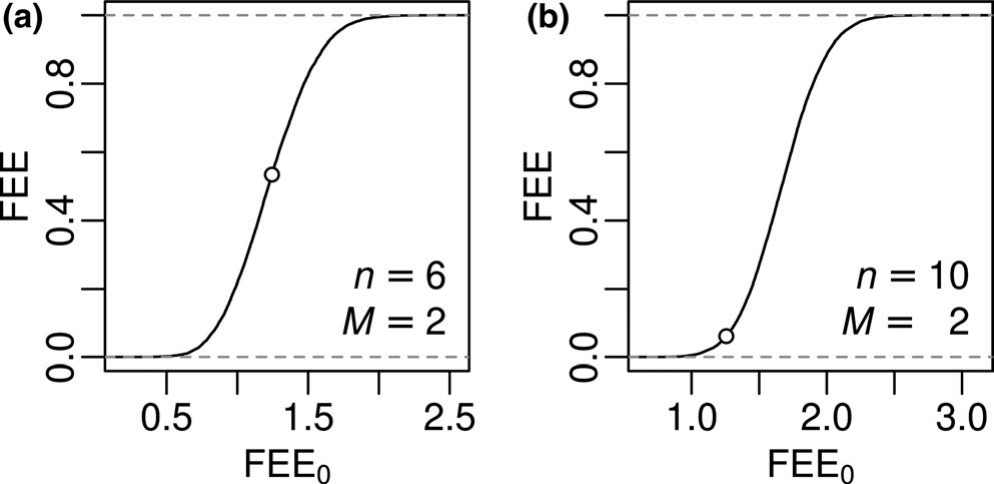
Examples of empirical cumulative distribution function curves ($F_{Q}$ in Equation 2) and illustration of FEE. The curves are derived from the FEE_0_ values of null model with a given species richness (*n*) and trait space dimension (*M*). Panels a and b here correspond to panels a and b in Figure 1, respectively, with open dots on each curve corresponding to the MSTs in Figure 1. Although FEE_0_ values are very similar in the two examples, their FEE values are quite different (Figure 1c). Note that the *x*‐axes (FEE_0_) in the two panels have different scales

FEE_0_ (Equation 1) combines both extension and evenness of the trait distribution into a single metric that is straightforward to calculate from the MST branch lengths. However, FEE_0_ is intrinsically dependent on species richness (*n*); that is, FEE_0_ exhibits statistical dependence on *n* across communities whose traits are randomly distributed in trait space. We thus do not recommend using FEE_0_ as a functional diversity index. We derived our functional extension and evenness (FEE) index by removing the intrinsic correlation between FEE_0_ and species richness. We accomplished this based on the cumulative distribution function of FEE_0_ values from a null model:

(2) $$\text{FEE}\equiv F_{Q}\left(X\right)=\frac{\sum_{i=1}^{Q}1\left\{x_{i}\leq X\right\}}{Q}$$

where $F_{Q}\left(X\right)$ is the empirical cumulative distribution function of FEE_0_ estimated from *Q* realizations of a null model of species assemblage (see below for description of the null model used in this paper); $x_{i}$ is the value of FEE_0_ from realization *i* of the null model; *X* is the observed value of FEE_0_ in the community of interest; and $1\left\{x_{i}\leq X\right\}$ is the indicator function (equal to 1 if $x_{i}\leq X$, and equal to 0 otherwise). In simple terms, FEE is the proportion of null model realizations that produce FEE_0_ values less than or equal to the observed FEE_0_. The general null hypothesis is that community assembly is a random sampling process from the species pool. In other words, to derive $F_{Q}\left(\cdot \right)$, one could generate many null communities of a given species richness by randomly sampling from the *M*‐dimensional trait space of the species pool. In this example, we assume no prior knowledge of the species pool, and we therefore implemented Equation (2) by drawing *Q* = 10,000 random samples from an *M*‐dimensional uniform(0,1) distribution (with all dimensions independent of each other). Each of these null communities has the same species richness (*n*) as the observed community of interest; that is, each null community is composed of *n* points randomly selected from an *M*‐dimensional unit hypercube. We then calculated FEE_0_ for each of these null communities to estimate the empirical cumulative distribution function of FEE_0_ for a given *n* (Figure 2). As Figure 2 shows, FEE_0_ is intrinsically dependent on species richness (*n*) and does not have a fixed upper limit as *n* varies. In contrast, FEE is expected to have no intrinsic dependence with *n* and is constrained to the 0–1 range, because it quantifies functional diversity relative to the null model distribution for a given *n*.

The FEE_0_ calculation in Equation (1) ignores the influence of species abundance in the functional diversity evaluation. Here, we propose a framework for adjusting the interspecies distances in trait space to account for species abundance differences. These adjusted distances are then used to identify a revised MST that accounts for species abundance, and this revised MST is used to calculate an abundance‐adjusted version of FEE_0_ (see details below). The null model analysis described above for FEE (Equation 2) can be used to translate the abundance‐adjusted FEE_0_ values into FEE values, because the same null model applies to the branch lengths of the abundance‐adjusted MST described below.

We consider the following principles in developing our distance adjustment strategy:

1. The purpose of adjusting distances in trait space is to adjust the influence of a given species pair on the MST structure (Figure 1). *Explanation:* As the distance between two species decreases, the likelihood that the pair will form an MST branch increases.
2. An adjusted distance (*dist_a_*) between two species in trait space should not be greater than the raw (unadjusted) distance (*dist*). *Explanation:* We assume that functional diversity is maximized when species abundances are equal. Therefore, accounting for abundance differences should not result in any increases in interspecies distances, which is equivalent to an extension of the occupied trait space relative to the equal abundance case. We impose the constraint *dist_a_* ≤ dist to avoid counterintuitive and logically inconsistent implications.
3. Adjusting the distance between two species in trait space does not imply adjustments to other distances. *Explanation:* Clearly, in a Euclidean space, adjusting the distance between two points (species) implies that the location of at least one point has changed, which would typically imply adjustments to other distances (if the community includes more than two species). However, we do not impose any such geometric constraints, and we consider adjustments for each interspecies distance independent of all other distances.

Following the three above principles, we propose two coefficients as the basis of a distance adjustment scheme for unequal abundances. The first coefficient addresses the summed abundance of a species pair, and the second coefficient addresses unevenness in abundances within a species pair. We use the following notation: *n* is the species richness of the community; the vector ***A*** refers to the set of species abundances in the community ($A_{1}$, $A_{2}$, … $A_{n}$); and the relative abundance of species *i* is $w_{i}=\frac{A_{i}}{\text{sum}\left(A\right)}$. We now describe the two coefficients, and then, we explain how they are combined to adjust interspecies distances in trait space.

*Coefficient for summed abundance of a species pair:* For a species pair with relative abundances $w_{i}$ and $w_{j}$, we calculate coefficient $K_{1}=\frac{2}{n}\cdot \frac{1}{w_{i}+w_{j}}$. Note that $K_{1}$ decreases as the summed relative abundance of the two species increases. The following three cases are illustrative: (a) If $w_{i}+w_{j}=2/n$ (as would occur if all species in the community have relative abundance 1/*n*), then $K_{1}=1$. (b) If the pair's total relative abundance, $w_{i}+w_{j}$, is greater than the average level (2/n), then $K_{1}<1$. (c) If $w_{i}+w_{j}<2/n$, then $K_{1}>1$.

*Coefficient for unevenness in abundance within a species pair:* We use the ratio of abundances between species *i* and *j*, $k_{\mathrm{ij}}$, to quantify unevenness in their abundances. We use the larger abundance ($w_{i}$ or $w_{j}$) as the numerator of the ratio, so that $k_{\mathrm{ij}}=\frac{\text{max}\left(w_{i},w_{j}\right)}{\text{min}\left(w_{i},w_{j}\right)}\geq 1$. We then calculate coefficient $K_{2}=\frac{2k_{\mathrm{ij}}}{k_{\mathrm{ij}}+1}$. Note that the minimum value of $K_{2}$ is 1, which occurs for two species of equal abundance (i.e., when $k_{\mathrm{ij}}$ is at its minimum value of 1). For species pairs with unequal abundance, $K_{2}$ increases as the abundance disparity ($k_{\mathrm{ij}}$) increases.

Combining these two coefficients, the abundance‐adjusted distance for a pair of species *i* and *j*, with locations $p_{i}$ and $p_{j}$ in trait space, is:

(3) $$dist_{a}\left(p_{i},p_{j}\right)=\min\left(K_{1}K_{2},1\right)\cdot dist\left(p_{i},p_{j}\right)=\min\left(\frac{4}{n\cdot \left(w_{i}+w_{j}\right)}\cdot \frac{k_{\mathrm{ij}}}{k_{\mathrm{ij}}+1},1\right)\cdot dist\left(p_{i},p_{j}\right)$$

where the minimum function, $\min\left(K_{1}K_{2},1\right)$, ensures compliance with Principle 2 above, which requires $dist_{a}\left(p_{i},p_{j}\right)\leq dist\left(p_{i},p_{j}\right)$.

Given the new set of adjusted interspecific distances from Equation (3), we calculate a new MST that minimizes the sum of adjusted distances. We then apply Equation (1) to this new MST to calculate the abundance‐adjusted FEE_0_. As noted above, the cumulative distribution function used to convert FEE_0_ to FEE (Equation 2) is unaffected by distance adjustments.

To understand how Equation (3) affects the MST structure (and thus FEE_0_), note the following: First, if all species in the community have equal abundance (i.e., all relative abundances are 1/*n*), then $dist_{a}\left(p_{i},p_{j}\right)=dist\left(p_{i},p_{j}\right)$. Second, consider a pair of species whose abundances are similar to each other and higher than the average abundance; that is, $w_{i}≅w_{j}>1/n.$ In this case, $K_{1}<1$ and $K_{2}≅1$, so the adjusted distance is smaller than the raw distance. This adjustment increases the likelihood that the pair forms an MST branch, as desired for a species pair of high abundance; that is, abundant species should have higher influence on the structure of MST and thus the quantification of functional diversity of the assemblage. Third, pairs of rare species have $K_{1}>1$ and $K_{2}\geq 1$, so that $\min\left(K_{1}K_{2},1\right)=1$. In this case, the raw distance remains unadjusted; this reduces the likelihood that pairs of rare species form MST branches, because their adjusted distances will be larger than for pairs of common species for whom $\min\left(K_{1}K_{2},1\right)<1$. Finally, note that there is a wide range of potential distance adjustments for pairs comprised of one common and one rare species. In this case, unequal abundance implies $K_{2}>1$; but, $K_{1}$ could be greater than or less than one, depending on the summed abundance of the two species.

Our distance adjustment scheme to account for species abundance differences is admittedly ad hoc and was developed through a lengthy trial‐and‐error process where we considered a variety of alternative schemes. We have tested the above scheme (Equation 3) under many simulated scenarios (e.g., see Results) and found its behavior to be logical and consistent. However, we have not rigorously proven that Equation (3) is optimal or that it would yield logical results in all possible scenarios. Indeed, there may be no single optimal scheme to integrate the consideration of abundance into the calculation of functional diversity (Kosman et al., 2021). We present our approach as a candidate for an abundance‐weighted distance scheme, and we hope that our paper stimulates future work on this topic.

### 2.5 Evaluating the FEE index

#### 2.5.1 *Relationships* *among indices*

We compared the behavior of FEE with functional diversity indices available in the FD package in R (Laliberté & Legendre, 2010; Laliberté et al., 2014), a widely used tool for calculating functional diversity indices (e.g., Barbaro et al., 2017; Ebeling et al., 2018; Gagic et al., 2015; Prado‐Junior et al., 2016; Price et al., 2014; Stuart‐Smith et al., 2013). The package provides functions to calculate several distance‐based functional diversity indices, including functional richness (FRic), functional evenness (FEve), functional divergence (FDiv), functional dispersion (FDis), and Rao's quadratic entropy (Q) (Botta‐Dukát, 2005; Laliberté & Legendre, 2010; Villéger et al., 2008).

To explore relationships among FEE, the indices listed above, and species richness, we generated a large number of artificial communities based on a null model of community assembly (random sampling from a trait space; see details below). In the null model, no external process or force acts on species assemblage. Thus, if an index is significantly correlated with species richness, we would conclude that the index is intrinsically correlated with species richness. For simplicity, we restricted this analysis to the presence–absence case (or, equivalently, the case where all species have equal abundance) in two‐dimensional trait space. We generated 200 artificial communities for each species richness value ranging from 2 to 100 (in total, 99 species richness levels × 200 replicates = 19,800 communities). Each of these 19,800 communities was randomly sampled from one of 19,800 independently generated species pools. Each of these pools contained 300 species whose two‐trait values were randomly selected from independent uniform(0,1) distributions. We used scatterplots and Pearson's *r* correlations to compare the diversity metrics to each other and to species richness.

#### 2.5.2 Scenario series tests

We evaluated the trends of FEE using the five artificial scenario series proposed by Schleuter et al. (2010) and used by Fontana et al. (2016). In these scenario series (T1 to T5, Figure S2, adapted from figure 2 in Schleuter et al. (2010)), species were defined by two traits (i.e., two‐dimensional trait space). Artificial communities were created by manipulating species composition with respect to species richness, abundance, and/or locations in the trait space. FEE was calculated for each scenario to evaluate whether its behavior across each series matched the behavior expected for a logically consistent functional diversity index. These series have previously been used to evaluate indices of functional richness, evenness, and divergence (Fontana et al., 2016; Schleuter et al., 2010). These previous evaluations revealed many inconsistencies in the behavior of some widely used indices, including cases where two indices focused on the same facet of functional diversity yielded inconsistent results for a given scenario series (Schleuter et al., 2010; Fontana et al., 2016; see also Table S1).

#### 2.5.3 Evaluation based on published criteria

We also evaluated our index against the criteria that Mason et al. (2003) and Ricotta (2005) summarized for functional diversity indices (see Table S2 for a list of these criteria). For those criteria that are straightforward to evaluate, we constructed artificial test scenarios (Figure S3) modeled after the scenario series of Schleuter et al. (2010). Criteria that are not relevant to our evaluation of FEE are also listed in Table S2, along with a brief explanation.

#### 2.5.4 Detecting community assembly processes

One potential application of functional diversity indices is to better connect ecosystem functioning and community ecology by revealing assembly processes that govern the distribution of functional traits (Götzenberger et al., 2012; Lamanna et al., 2014; Mouchet et al., 2010). Three widely studied assembly processes are neutral dynamics, environmental niche filtering, and limiting similarity. Neutral community assembly is based on the assumption that all individuals in a community are ecologically equivalent, such that species composition and abundances are solely the results of stochastic processes unrelated to functional traits (Hubbell, 2001). In contrast, environmental niche filtering (e.g., stress tolerance) increases species similarity through abiotic constraints that only allow certain traits to persist (Cornwell et al., 2006; Weiher & Keddy, 1995; Zobel, 1997). Finally, limiting similarity (i.e., niche partitioning and competitive exclusion) prevents very similar species from stably coexisting (Chesson, 2000; Hardin, 1960; Macarthur & Levins, 1967; Spasojevic & Suding, 2012).

To investigate the performance of FEE in discriminating among community assembly processes, we generated artificial communities according to three different assembly processes (neutral, niche filtering, and limiting similarity), and we evaluated the capacity of FEE and some other indices to correctly diagnose the assembly processes. We used the R package “ecolottery” (Munoz et al., 2018) to create these artificial communities by simulating community dynamics from random initial compositions. We generated 10,000 independent species pools and their initial compositions in one‐dimensional trait space. Each of these species pools contained 200 species whose trait values were randomly drawn from a uniform(0,1) distribution. An initial community was generated from each pool by randomly selecting 30 species, each with an initial abundance of 5 individuals. Starting from each of these 10,000 initial conditions, we used the “forward” function in the “ecolottery” R package (Munoz et al., 2018) to simulate three different community assembly trajectories, one for each assembly process: neutral, niche filtering (NF), and limiting similarity (LS). Each simulation lasted 150 generations. In each time step, 2% of individuals died; and each dead individual had an 80% chance of being replaced by local reproduction and a 20% chance of being replaced by immigration.

We calculated FEE and other indices available in the FD package (Laliberté & Legendre, 2010; Laliberté et al., 2014) for the simulated communities, and we quantified the relationship between each index and the three assembly processes. The expected rank order of functional diversity among the three processes is limiting similarity > neutral assembly > niche filtering (Mouchet et al., 2010). We used paired *t* tests to evaluate these expectations for each index. In addition, we used linear regression to evaluate the influence of species richness on each diversity index under each assembly process (Pavoine & Bonsall, 2011). We conducted these tests for both the presence–absence and species abundance versions of FEE.

## 3 RESULTS

### 3.1 Relationships among indices

**FIGURE 3:**
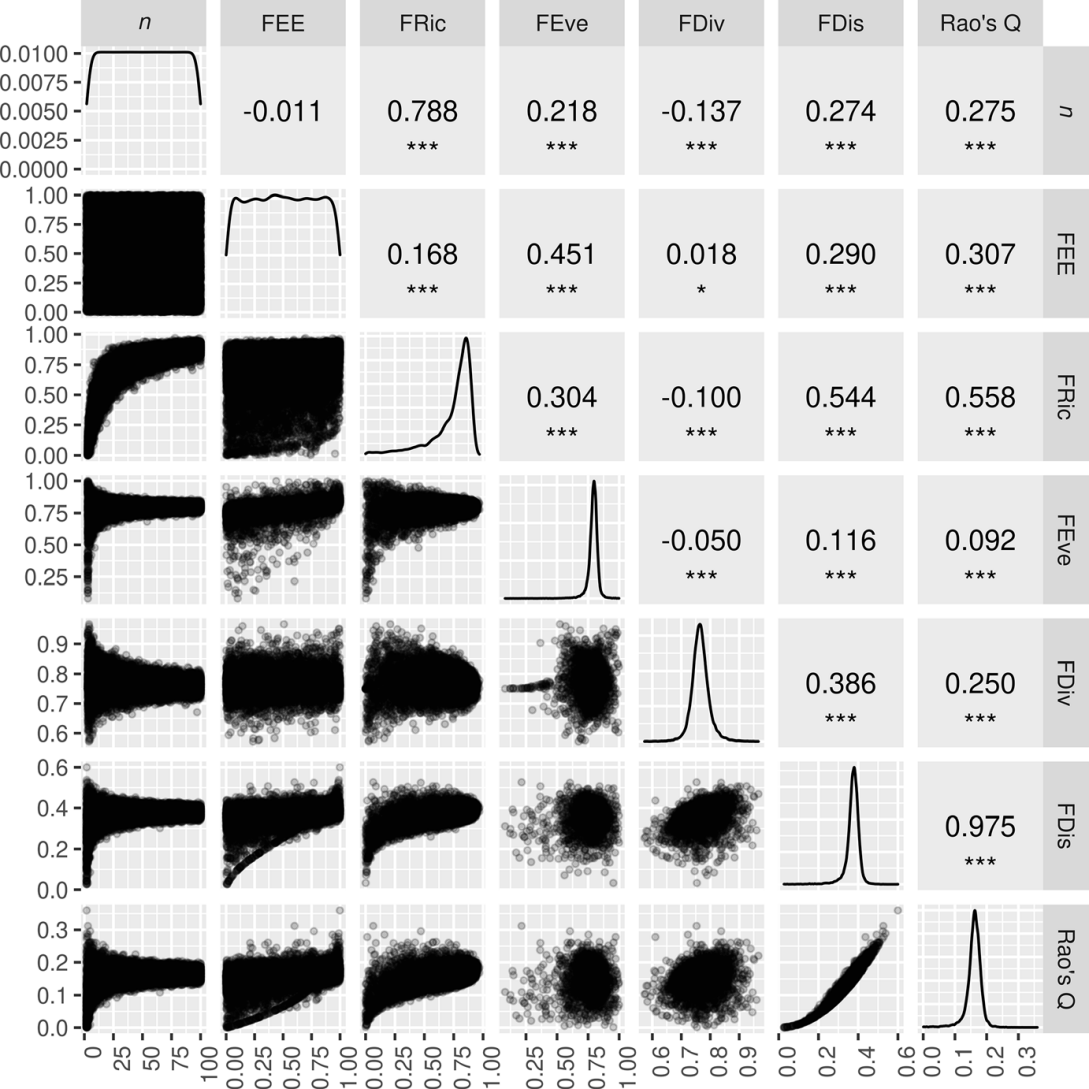
Pairwise scatter plots (lower left) and Pearson's *r* correlations (upper right) among species richness (*n*) and functional diversity indices (including our FEE index and FD indices: FRic, FEve, FDiv, FDis, and Rao's Q) for data generated by a null model (random species distribution in two‐dimensional trait space; 200 replicate communities for each species richness value from 2 to 100). The diagonal cells show the distributions of the corresponding indices. Significance levels: ***, <0.001; *, <0.05; no asterisk, ≥0.05

Pairwise scatter plots for FEE, five indices from the R package FD (Laliberté & Legendre, 2010; Laliberté et al., 2014), and species richness are shown in Figure 3. The five FD indices are FRic (Functional Richness), FEve (Functional Evenness), FDiv (Functional Divergence), FDis (Functional Dispersion), and Rao's Q (Rao's Quadratic entropy). FEE was nearly independent of FDiv (*r* = 0.018) and showed a weak to moderate positive correlation with the other four FD indices (*r* ranging from 0.168 to 0.451) (Figure 3). As expected, FEE was not significantly correlated with species richness (*n*). FDiv had a weak negative correlation with *n* (*r* = −0.137), and the remaining indices (especially FRic) were positively correlated with *n* (Figure 3). Finally, the five FD indices were often correlated with each other, with the highest correlation (*r* = 0.975) for FDis and Rao's Q (Figure 3).

There are some minor differences between results in Figure 3 and those reported in Villéger et al. (2008). For example, Villéger et al. (2008) found that FDiv was not correlated with species richness (*n*), whereas our results showed a weak negative correlation between FDiv and *n*. Also, Villéger et al. (2008) reported that the three indices they studied (FRic, FDiv, and FEve) were not correlated with each other, whereas our results show they are all weakly but significantly correlated with each other (especially FRic and FEve). One possible reason for these inconsistencies is the different species richness range in their study (10–40) compared with ours (2–100). As the scatter plots with *n* show, many indices show high variance and/or different trends at low values of species richness (e.g., 2–10 species) (Figure 3). When we restricted our analysis to the 10–40 species richness range, our results (not shown) were more similar to those of Villéger et al. (2008). Another reason for apparent discrepancies between our study and Villéger et al. (2008) is the large sample of artificial communities we analyzed (19,800), which provides high statistical power to detect weak relationships. Other studies (e.g., Mouchet et al., 2010) that used methodology and a species richness range similar to ours reported a significant correlation between FRic and FEve (Spearman's *ρ* = 0.285), consistent with our results.

### 3.2 Scenario series tests

The behavior of FEE in the scenario series tests proposed by Schleuter et al. (2010) (T1 to T5, Figure S2) was consistent with expectations (Table S1). We summarize and explain these trends below. Table S1 presents the expected trends for three diversity facets (functional richness, evenness, and divergence) according to Schleuter et al. (2010) and Fontana et al. (2016), and summarizes observed trends (our test results) for FEE and the five other FD indices in Figure 3. Below, we use the terms “richness,” “evenness,” and “divergence” to refer to these concepts in general, rather than as labels for specific indices (e.g., FRic, FEve, and FDiv).

In T3, where richness, evenness, and divergence are nonincreasing from left to right in Figure S2 (richness remains constant; the other two facets of diversity decrease), FEE also decreases from left to right. In T4, FEE increases from left to right in Figure S2, consistent with richness, evenness, and divergence (all are nondecreasing from left to right).

In T5, FEE increases from left to right in Figure S2, which is mostly consistent with the expectations of Schleuter et al. (2010). The main discrepancy concerns the far‐left scenario, which Schleuter et al. (2010) considered as having the highest evenness (Table S1). However, we consider the evenness of the first scenario in T5 as the lowest among the four scenarios in T5 for the following reasons. First, all T5 scenarios have the same species and functional richness; but the disparity of abundance among species is greatest in the first scenario (far‐left in Figure S2: 50 for one species and 1 for the other 24 species). In contrast, the other T5 scenarios have abundances of 25 for two species and 1 for the other 23 species. Shannon's H or its derived evenness (equitability) index also identifies the first T5 scenario as having the lowest evenness. Second, the first T5 scenario has the most centralized distribution in trait space, and in this sense, it has the lowest evenness as well.

The T1 and T2 series are more complicated because different facets of functional diversity have inconsistent expected trends (Table S1). Even for a given facet, expectations may differ among researchers; for example, there are minor differences in expectations between Schleuter et al. (2010) and Fontana et al. (2016) for some series (Table S1). Not surprisingly, FEE's trend for T1 and T2 is consistent with expectations for some but not all facets of diversity. For example, FEE's trend for T1 and T2 is consistent with the expected richness trends of Fontana et al. (2016), and with expectations for divergence for scenarios 2–4 (three right‐most in Figure S2) of both Schleuter et al. (2010) and Fontana et al. (2016). However, these trends are not consistent with the expected evenness trends (Table S1).

In summary, FEE trends in the T1‐T5 test scenarios are mostly consistent with previously published expectations for functional richness, evenness, and divergence, but complete consistency is not possible when expected trends for the three facets differ. FEE performed favorably compared with the other five FD indices, which did not always show trends consistent with those expected for their corresponding diversity facet (Table S1).

### 3.3 Evaluation based on published criteria

Table S2 details our evaluation of FEE with respect to the criteria proposed by Mason et al. (2003) and Ricotta (2005). Except for a few criteria that are not relevant for FEE (i.e., those marked “N/A” in Table S2), the FEE index appears to fulfill most of these criteria. Our evaluation is necessarily informal, as methods to rigorously evaluate indices against the criteria are not available. In contrast, to FEE, other FD indices fail to fulfill the criteria in a number of cases (Table S2).

### 3.4 Detecting community assembly processes

FEE values for artificial communities generated under different assembly processes tended to be consistent with the expected order: limiting similarity (LS) > neutral > niche filtering (NF) (Mouchet et al., 2010). These results held for FEE both with and without considering species abundance (Table 1). The FEE index outperformed the other FD indices in detecting community assembly processes, especially in discerning neutral and LS processes (Table 1).

**TABLE 1:** Paired *t* tests to evaluate the performance of functional diversity indices in detecting the three community assembly processes: neutral, niche filtering (NF), and limiting similarity (LS)

H1 of paired *t* test	FEE		FRic	FEve		FDis		Rao's Q	
	p‐a	ab.	p‐a or ab.	p‐a	ab.	p‐a	ab.	p‐a	ab.
Neutral > NF	70.0***	45.8***	72.3***	34.8***	12.2***	195.6***	109.3***	195.6***	109.0***
LS > neutral	22.4***	17.9***	2.1*	1.9*	−14.7	7.0***	−0.2	9.7***	2.8**

Two hypotheses are tested (Mouchet et al., 2010): functional diversity is higher under neutral assembly than under niche filtering (neutral > NF), and functional diversity is higher under limiting similarity than under neutral assembly (LS > neutral). Artificial community data were simulated in a one‐dimensional trait space. The community data were analyzed both as presence–absence (p‐a) data and as abundance (ab.) data. The table shows the one‐sided *t*‐statistics and their associated significance levels (***, < 0.001; **, < 0.01; *, < 0.05; no asterisk, ≥ 0.05). FDiv index is not included here because it is not available for the one‐trait case (Laliberté et al., 2014).

**FIGURE 4:**
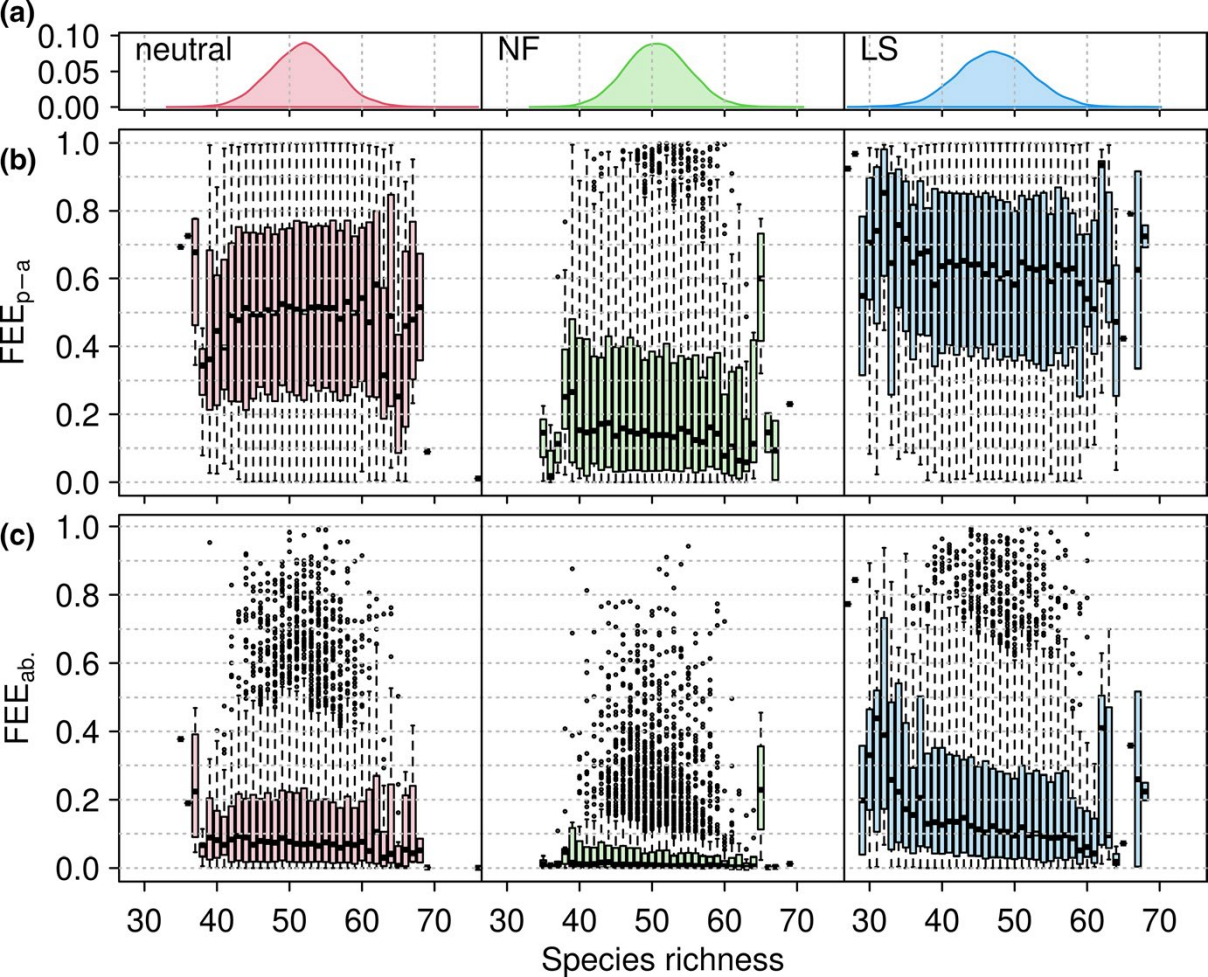
FEE under three community assembly processes: neutral, niche filtering (NF), and limiting similarity (LS). Distributions of species richness (panel a) under the three assembly processes differ in mean value. Boxplots of FEE for the presence–absence (p‐a) case (panel b) and the abundance (ab.) case (panel c) show that the order of functional diversity among the three processes is LS > neutral > NF (see also Table 1), as expected for a well‐behaved functional diversity index (Mouchet et al., 2010). Boxplots also show that the relationship between FEE and species richness differs among different assembly processes (see also Table S3). The colored boxes in the boxplots (panels b and c) show the interquartile range, the dashed whiskers show 1.5 times the interquartile limits, and the points (if any) show outliers

Although the FEE index is designed to be intrinsically independent of species richness (see Methods), as confirmed by null model tests (Figure 3), relationships between FEE and species richness may arise under nonrandom community assembly. For neutral assembly, FEE was not significantly correlated with species richness in either the presence–absence case (Figure 4b) or the abundance‐weighted case (Figure 4c). For the NF and LS assembly processes, FEE decreased as species richness increased (Figure 4 and Table S3). The other indices showed a variety of relationships with species richness under the different assembly processes (Table S3). Rao's Q showed different correlations with species richness under different assembly processes (insignificant for neutral, negative for NF, and positive for LS; Table S3).

## 4 DISCUSSION

Our FEE index is based on a simple and straightforward notion: A community of high diversity is one whose species are distant from each other in trait space, in terms of both overall extent and evenness of spacing. The simple, intuitive notion upon which FEE is based avoids some counterintuitive behaviors of other functional diversity indices (e.g., Figure S1) and leads to desirable properties (e.g., Table S2). Our FEE index was positively correlated with other indices in most cases (Figure 3), but these correlations were weak or moderate in strength (*r* < 0.5), suggesting that FEE provides information that is not captured by individual single‐facet indices. In all tests, we conducted (including evaluations based on published test scenarios and criteria; Tables S1 and S2) FEE performed as expected for a well‐behaved functional diversity index and generally outperformed other widely used indices that we tested. Furthermore, some other indices have certain data requirements that restrict their use (see Introduction, also Fontana et al., 2016; Schleuter et al., 2010). The FEE index overcomes these constraints.

### 4.1 Functional diversity versus species richness

As described in Introduction, independence from species richness is considered a desirable property for a functional diversity index (Mason et al., 2003; Pavoine & Bonsall, 2011; Pavoine et al., 2013; Petchey & Gaston, 2006; Schleuter et al., 2010; Villéger et al., 2008). Not surprisingly, indices of functional richness are often correlated with species richness (Schleuter et al., 2010). Our results show that some popular indices of functional evenness and dispersion (e.g., FEve, FDis, and Rao's Q) also depend on species richness to some degree (Figure 3). If a functional diversity index is intrinsically correlated with species richness, it is difficult (or impossible) to identify the specific contribution of the traits included in the index calculation to ecosystem function. Many studies ignore the possible confounding effect of taxonomic diversity when comparing functional diversity across communities of varied species richness. The robustness of conclusions drawn from such analyses is questionable (Mason et al.,. 2003, 2013; Mouchet et al., 2010; Schleuter et al., 2010).

To remove the intrinsic dependence of our index on species richness, we used the cumulative density function of the raw FEE_0_ metric from a null model analysis (with a given species richness) to calculate FEE (which reflects the location of the observed FEE_0_ value within the null distribution). Null models offer a promising approach to standardizing functional diversity indices when comparing communities that differ in species richness (Bernard‐Verdier et al., 2012; Mason & de Bello, 2013; Schleuter et al., 2010). In our analyses that were designed to explore the properties of FEE, we assumed no prior knowledge of the trait distribution of the species pool in the null model, and our null model thus entailed random sampling from a uniform trait distribution. In real‐world applications, it may be preferable to base the null model (and thus the cumulative distribution function, Equation 2) on the observed trait distribution of the species pool (or the combination of regional species pools if comparing functional diversity across regions). Because the definition of species pool can influence index values and thus potentially affects inference of assembly processes (Lessard et al., 2012), it is important to define a single species pool for a given study (e.g., when using FEE to compare functional diversity across different communities or regions).

Because the FEE index is intrinsically independent of species richness, we suggest that it allows for fair comparisons of functional diversity across communities that differ in species richness. However, the intrinsic independence property does not imply that FEE is uncorrelated with species richness in the presence of certain community assembly processes. Our results showed FEE to be independent of species richness under neutral community assembly, but not under other community assembly processes (limiting similarity and environmental niche filtering; Figure 4). Thus, the relationship between FEE and species richness might provide a useful tool to diagnose the presence of non‐neutral assembly processes. Other single‐facet indies (e.g., Rao's Q) might have this potential as well (Table S3). Before using other indices in this context, it may be helpful to first remove any intrinsic dependence on species richness (e.g., using the null model approach). Because the tests presented here were based on artificial communities, and reality is more complicated than a simple one‐to‐one relationship between assembly processes and species coexistence patterns (e.g., Cadotte & Tucker, 2017; Caruso et al., 2011; Mayfield & Levine, 2010), further investigation of the FEE versus species richness relationship as a potential diagnostic tool warrants further investigation.

The discussion above is not intended to deny the usefulness of indices that are intrinsically correlated with species richness. The choice of functional diversity indices should be guided by the research questions. For example, if a study focuses on comparing compositional similarity of traits among assemblages (rather than comparing their functional diversity levels), approaches based on Hill numbers (Chiu & Chao, 2014) might be appropriate even though functional Hill numbers are intrinsically correlated with taxonomical diversity (Vega‐Álvarez et al., 2019). A major advantage of Hill numbers is that they can be used to decompose a gamma diversity into alpha and beta diversities (Chao et al., 2014). However, in cases where it is desirable to disentangle the effect of species richness from the “pure” effect of functional diversity, a richness‐independent functional diversity index would be preferred.

### 4.2 Applications and extensions

FEE is a distance‐based index in which dissimilarity between a pair of species is represented by their distance in trait space. In this study, we focused on continuous traits and used Euclidean distance to quantify species dissimilarity. Other trait types and distance calculation schemes, which can be used with existing distance‐based indices (e.g., those in the FD R package, Laliberté et al., 2014), should all be applicable to the FEE index. For example, principal coordinate analysis (PCoA) could be applied to the raw trait space, and interspecific distances could then be calculated in the trait space defined by the PCoA axes (Laliberté & Legendre, 2010). Approaches for analyzing qualitative traits adopted by some common distance‐based indices (e.g., FD indices in Laliberté et al., 2014) can also be implemented with FEE. It is possible that certain distance or dissimilarity schemes (including when species abundance is considered, as in Equation 3) could result in nonmetric distance matrices (Gower & Legendre, 1986); however, as long as a distance scheme can be applied to identify MST (i.e., the distance matrix for a community is complete), we expect that the FEE index would perform well, as in the tests presented here.

Given its desirable properties, we suggest that FEE could be usefully applied in a variety of contexts related to the functional diversity of ecosystems, including studies examining the role of functional diversity in ecosystem services, productivity, and/or resilience. Two features of FEE make it especially well suited to broad‐scale studies spanning communities that vary widely in species richness and that may share few if any common species: (a) FEE quantifies diversity for all communities in a consistent frame of reference (the trait distribution of a common species pool); and (b) FEE is intrinsically independent of species richness, allowing for fair comparisons of functional diversity across broad environmental gradients. While the FEE index offers certain advantages, we do not suggest that it alone provides a complete view of functional diversity. Depending on the goals of an analysis, FEE might be used along with other functional diversity indices, as well as taxonomic and phylogenetic diversity indices, to provide a more complete picture of biodiversity.

Our study is limited to considering interspecific trait variation, but there is also growing interest in quantifying intraspecific trait variation and its role in functional diversity (Fontana et al., 2016). Thus, one avenue for future work would be to explore extending the framework developed here to include intraspecific trait variation. In principle, the FEE index could be applied to individuals (rather than species) based on identifying the minimum spanning tree (Figure 1) of individuals in trait space.

The index calculations and other analyses in this study were conducted in R. It is expected that as species richness and/or trait number increase, calculation time would increase as well. Based on our experiments, calculation time–cost for the FEE index is at a similar level as many widely used indices.

## 5 CONCLUSIONS

Our proposed functional extension and evenness (FEE) index is based on the straightforward notion that a community of high diversity is one whose species are distant from each other in trait space. The FEE index has the following properties: (a) the index is intrinsically independent of species richness, and thus allows fair comparisons of functional diversity across different species richness levels; (b) it overcomes data constraints of some other functional diversity indices (e.g., number of traits, species richness, and singularity of trait values); (c) it considers the species pool rather than only the community itself in defining the trait space and null models, thereby allowing for standardized comparisons across communities; (d) it shows promise in diagnosing community assembly processes; and (e) it performs well in regard to previously published tests and criteria for functional diversity indices. Collectively, these attributes highlight the potential of FEE as a convenient and reliable tool for quantifying the functional diversity of ecosystems.

## CONFLICT OF INTEREST

We confirm that we have no conflict of interest in the subject matter discussed in this manuscript.

## AUTHOR CONTRIBUTIONS

Tao Zhang: conceived of the conceptual ideas, developed the theoretical formalism and methods, performed the analyses and computations, drafted the manuscript. Grant M. Domke: co‐supervised the project, discussed the ideas, edited the manuscript. Matthew B. Russell: co‐supervised the project, discussed the ideas, edited the manuscript. Jeremy W. Lichstein: contributed to conceptualization and theoretical formalism, discussed the ideas and results, revised and edited the manuscript.

### OPEN RESEARCH BADGES

This article has earned an Open Data Badge and Open Materials Badge for making publicly available the digitally‐shareable data necessary to reproduce the reported results.

## Data Availability

Data and code are archived in Dryad and publicly available (https://doi.org/10.5061/dryad.b8gtht7c7). The data include empirical cumulative distribution functions for the FEE index, and the simulated species traits and abundances used in our analyses. The R code performs all analyses in our paper, including FEE calculations for the simulated communities, statistical analyses, and generating figures.
