# Supplementary material for: An index for measuring functional extension and evenness in trait space
Source: Ecol Evol. 2021 May 6;11(12):7461–73. doi: 10.1002/ece3.7577 (PMC8216966; doi:10.1002/ece3.7577)
Supplement: Supplementary file 1 [file ECE3-11-7461-s001.pdf]

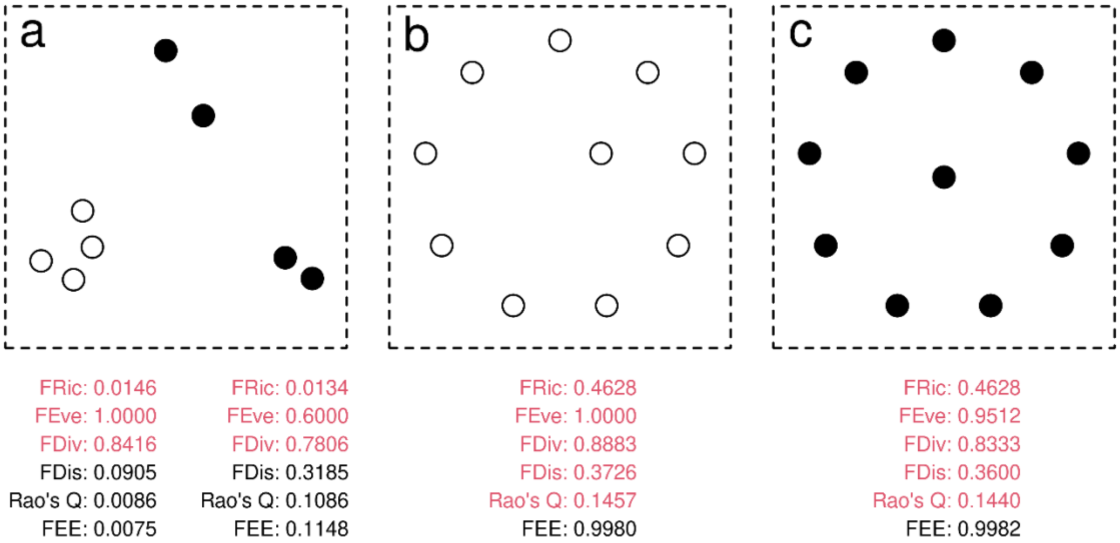

Figure S1. Examples of counter-intuitive results for some popular functional diversity indices, calculated with the FD package in R (Laliberté et al. 2014). The dashed boxes indicate the 2-dimensional trait space (unit square). Intuitively, between the two communities (white and black) in panel *a*, the ‘black’ community has higher functional diversity than the ‘white’ one. However, the three representative indices of functional richness, evenness, and divergence (FRic, FEve, and FDiv) imply the opposite relationship (panel *a*). Between the panels *b* and *c*, all species are same except the one in the center. Compared to panel *c*, the trait space in panel *b* is not evenly taken by the central species. Thus, functional diversity in panel *b* is expected to be slightly lower than in panel *c*. However, none of the five functional diversity indices we tested show this expected relationship. In the three panels, indices showing counter-intuitive results are highlighted in red. In contrast to the counter-intuitive results shown in the figure, values of the FEE index proposed in this paper are consistent with intuition.

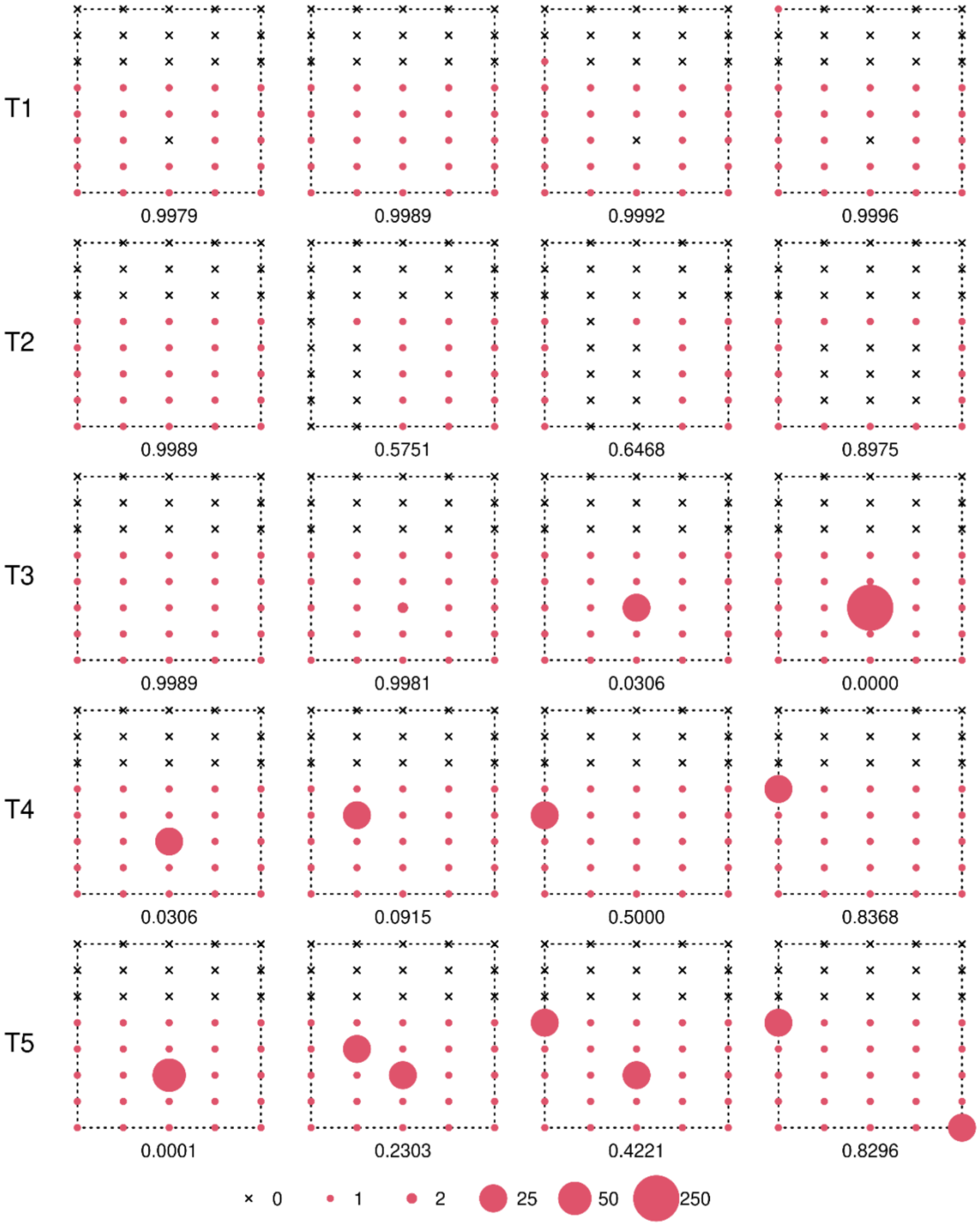

Figure S2. Five scenario series tests (T1 to T5) with artificial communities proposed by Schleuter et al. (2010) to their evaluate functional diversity indices. The species pool contains 40 species (black crosses and red dots). Red dots show the species presented in a community, and their size represents species abundance. The dashed box in each panel presents the 2-dimensional trait space (unit square). Values of our FEE index, which accounts for species abundances (Equation 3), are given under each panel. Table S1 summarizes the trends of indices (FEE and five FD indices) for each series, and the expected behavior of three components of functional diversity according to Schleuter et al. (2010) and Fontana et al. (2016).

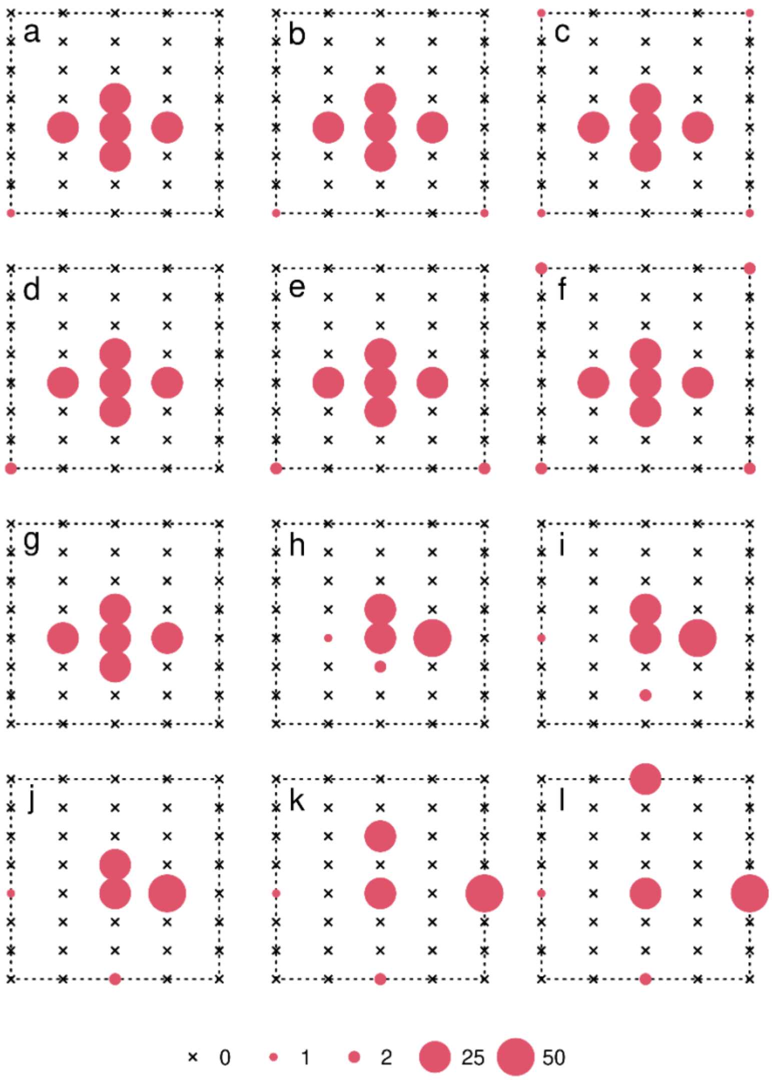

	Left	Middle	Right
	a	b	c
FRic	0.1429	0.3571	1.0000
FEve	0.5645	0.5500	0.6049
FDiv	0.5920	0.4951	0.2953
FDis	0.1621	0.1663	0.1747
Rao's Q	0.0363	0.0394	0.0478
**FEE**	**0.1154**	**0.1853**	**0.7986**
	d	e	f
FRic	0.1429	0.3571	1.0000
FEve	0.5762	0.5594	0.6106
FDiv	0.5965	0.5058	0.3183
FDis	0.1670	0.1751	0.1911
Rao's Q	0.0394	0.0454	0.0615
**FEE**	**0.1194**	**0.1983**	**0.8172**
	g	h	i
FRic	0.0714	0.0714	0.1607
FEve	0.8182	0.7246	0.5626
FDiv	0.6790	0.6581	0.6520
FDis	0.1571	0.1425	0.1475
Rao's Q	0.0332	0.0211	0.0249
**FEE**	**0.0694**	**0.0362**	**0.2035**
	j	k	l
FRic	0.2143	0.3571	0.5000
FEve	0.5332	0.6810	0.7869
FDiv	0.6595	0.6723	0.6868
FDis	0.1501	0.2874	0.3487
Rao's Q	0.0270	0.0869	0.1330
**FEE**	**0.2927**	**0.8329**	**0.9711**

Figure S3. Tests with artificial communities to evaluate FEE (accounting for species abundances according to Equation 3) and other single-facet indices with respect to the criteria summarized by Mason et al. (2003) and Ricotta (2005). Evaluation results are summarized in Table S2. The species pool contains 40 species (black crosses and red dots). Red dots show the species present in a community, and their size represents species abundance. The dashed box in each panel represents the 2-dimensional trait space (unit square). Values of our FEE index (in bold) and the single-facet indices are given in the table to the right of the figure.

Table S1. Expected and observed trends for functional diversity indices for five scenario series tests (T1-T5 in Figure S2), adapted from Figure 2 in Schleuter et al. (2010). Symbols in the table summarize the expected trends for three functional diversity components (richness, evenness, and divergence) according to Schleuter et al. (2010) (blue symbols) and Fontana et al. (2016) (red symbols), and observed trends (black symbols) for five single-facet indices (FRic, FEve, FDiv, FDis, and Rao's Q), and FEE. Expected trends from Schleuter et al. (2010) for T1-T5 are from their Table 2, and those from Fontana et al. (2016) for T1-T2 are from their Table 1. All black symbols show observed trends from our analysis. FRic, FEve, and FDiv, respectively, are compared to the expected trends for richness, evenness, and divergence (blue and red symbols). No expected trends for FDis and Rao's Q are available, but their observed trends for T1-T5 are presented for completeness. Actual values for FEE are shown in Figure S2 (actual values for other indices are not shown, but are summarized qualitatively in this table). The order of symbols in each list (from top to bottom) corresponds to the scenarios (from left to right) in each series in Figure S2. Symbols: ∘ indicates the diversity of the reference community in each series (left column in Figure S2), or communities whose diversity is equal to that of the reference community; + indicates that diversity is higher than in the reference community; − indicates that diversity is lower than in the reference community. Following the notation of Schleuter et al. (2010) and Fontana et al. (2016), the number of + or − symbols indicates the magnitude of difference relative to the reference community.

Scenario series	Functional richness			Functional evenness			Functional divergence			FDis	Rao's Q	FEE
T1	∘	∘	∘	∘	∘	∘	∘	∘	∘	∘	∘	∘
	+	+	∘	+	+	++	−	−	−−−	−	−	+
	+	++	+	−	−	+	+	+	−	+	+	++
	+	+++	++	−−	−−	−	++	++	−−	++	++	+++
T2	∘	∘	∘	∘	∘	∘	∘	∘	∘	∘	∘	∘
	−	−−−	−	−	−	−	−	−	+	−	−	−−−
	−	−−	∘	−−−	−−−	−−−	+	+	++	+	+	−−
	−	−	∘	−−	−−	−−	++	++	+++	++	++	−
T3	∘		∘	∘		∘	∘		∘	∘	∘	∘
	∘		∘	−		−	−		−	−	−	−
	∘		∘	−−		−−	−−		−−	−−	−−	−−
	∘		∘	−−−		−−−	−−−		−−−	−−−	−−−	−−−
T4	∘		∘	∘		∘	∘		∘	∘	∘	∘
	∘		∘	∘		∘	+		+	+	+	+
	∘		∘	+		∘	++		++	++	++	++
	∘		∘	++		+	+++		+++	+++	+++	+++
T5	∘		∘	∘		∘	∘		∘	∘	∘	∘
	∘		∘	−−−		−−−	+		+	+	+	+
	∘		∘	−−		−−	++		++	++	++	++
	∘		∘	−		−	+++		+++	+++	+++	+++

Table S2. Evaluation of our FEE index and some single-facet indices with respect to the criteria proposed by Mason et al. (2003) (criteria 1-10 in our table) and Ricotta (2005) (criteria 11-14 in our table). Evaluation results in the table are informal (rigorous and general proofs are not available) and are mainly based on the results presented in Figure S3. FEE results in this table and Figure S3 account for species abundances according to Equation (3). In the 'Result' column, 'S' indicates satisfied and 'N/A' indicates that the criterion is not applicable to FEE. In the 'Notes' column, text in square brackets refers to previously published indices. All other text in the 'Notes' column refers to our FEE index.

Criterion from the literature	Result	Note
1. Be constrained to the 0-1 range (for convenience) and use that range well.	S	See the relevant distributions in Figure 3.<br>[Rao's Q only occupies a small part of the range in our tests (Figure 3).]
2. Reflect the range of character values present, since that is the point of the index.	S	
3. Reflect the contribution of each species in proportion to its abundance; a community is not functionally diverse if all species with extreme trait values are rare.	S	Comparing Figure S3*a* and S3*g* (or Figure S3*a* and S3*b*) shows that introducing a rare species on the edge of trait space has only a small effect. Thus, FEE satisfies this criterion in a broad sense (if not strictly 'in proportion' to abundance).<br>[FRic and FEve do not meet this criterion.]
4. Decrease when the abundance of a minor species with an extreme trait value decreased.	S	See difference between Figure S3*a* and S3*d* (or between Figure S3*b* and S3*e*, or between Figure S3*c* and S3*f*).<br>[FRic does not meet this criterion.]
5. Not change appreciably when a very rare species disappears.	S	Comparing Figure S3*a* and S3*g* (or Figure S3*a* and S3*b*) shows that removing rare species has only a small effect. However, in these comparisons, the effect is noticeable because the removed species occupied a distinct part of trait space.<br>[FRic does not meet this criterion well.]
6. Be unaffected by the units in which the trait is measured. This is essential for traits that could be measured on more than one scale (e.g., mm, cm, or m).	S	Trait values in the species pool are normalized to the 0-1 range.
7. Be symmetrical with regard to small and large character values.	S	Using the cumulative distribution function from a null model to translate FEE_0 to FEE (Equation 2) leads to a broad and symmetric distribution (see the probability density function of FEE in Figure 3).<br>[FRic, FEve, FDiv, FDis, and Rao's Q do not meet this criterion.]
8. Be unaffected by the units in which the abundance is measured. It is unacceptable to have the index value dependent on the unit chosen (e.g., mg, g, or kg).	S	The abundance-adjusted version of FEE relies on unitless relative-abundance weights (Equation 3). The definition of these weights is flexible; e.g., they could be based on biomass, percent cover, individual density, etc.
9. Be unaffected by species richness. The number of taxonomic species per se is not relevant to functional diversity.	S	FEE is intrinsically independent of species richness (n) (Figure 3); i.e., FEE is independent of n when communities are randomly sampled from the species pool, although FEE may be correlated with n under some assembly processes (Figure 4 and Table S3).<br>[FRic, FEve, FDiv, FDis, and Rao's Q do not strictly meet this criterion, although some of these are only weakly correlated with n; see Figure 3.]
10. Be unaffected when a species is split in two (i.e., one species is replaced by two species with the same traits and the same total abundance as the original).	N/A	We adopt the 'functional species' concept of Ricotta (2005), such that two species with identical trait values would be considered the same species.
11. Set monotonicity (a subset of a community is less diverse than the entire community).	N/A	This criterion focuses on species or functional richness, which is not applicable to indices (such as FEE) that account for abundance (Ricotta

Criterion from the literature	Result	Note
		2005) or trait distribution factors (e.g., evenness, divergence, dispersion, etc.) (Villéger et al. 2008, Laliberté and Legendre 2010).
12. Twinning (diversity should not be increased by the addition of a species that is functionally identical to a species already in the set).	N/A	This criterion is not applicable to indices that account for abundance (Ricotta 2005). If we consider the presence-absence version of FEE, then the criterion is satisfied (see Notes for #10 above).
13. Monotonicity in distance (if distances between some species increase, diversity does not decrease).	S	See trend from Figure S3*h* to S3*l*.<br>[FEve and FDiv do not meet this criterion.]
14. Concavity (the average diversity of a set of communities is less than the diversity of the aggregated pool).	N/A	See Notes for #11 above.

Table S3. The coefficients ($\times 10^{-4}$) of species richness in the linear regression models of functional diversity indices under the three community assembly processes: neutral, niche filtering (NF), and limiting similarity (LS). Artificial community data were analyzed both as presence-absence (p-a) data and abundance (ab.) data. The table shows the coefficients and their associated significance levels (***, < 0.001; **, < 0.01; *, < 0.05; no asterisk, ≥ 0.05). Boxplots for the FEE results are shown in Figure 4*b*-*c*. The FDiv index is not included here because it is unavailable for the one-trait case (Laliberté et al. 2014).

Assembly process	FEE		FRic	FEve		FDis		Rao's Q	
	p-a	ab.	p-a or ab.	p-a	ab.	p-a	ab.	p-a	ab.
neutral	7.569	-8.276	6.877 ***	1.493 *	10.60 ***	0.455	1.424 *	0.139	0.576
NF	-21.19 ***	-14.90 ***	7.573 ***	0.135	5.851 ***	-1.274 **	-3.541 ***	-0.854 ***	-1.874 ***
LS	-21.21 ***	-35.27 ***	8.696 ***	0.707	12.04 ***	2.834 ***	6.553 ***	1.548 ***	3.309 ***
